# Mounier-Kuhn Syndrome: Anesthetic Experience

**DOI:** 10.1155/2012/674918

**Published:** 2012-04-03

**Authors:** Deepu Sasikumaran Ushakumari, Navneet Grewal, Michael Green

**Affiliations:** ^1^Drexel University College of Medicine and Hahnemann University Hospital, Philadelphia, PA 19102, USA; ^2^Department of Anesthesiology, Drexel University College of Medicine and Hahnemann University Hospital, New College Building, Room 7502, Philadelphia, PA 19102, USA

## Abstract

Mounier Kuhn syndrome, or congenital tracheobronchomegaly, is an under diagnosed clinical entity with peculiar anatomical and physiological features making anesthetic care challenging. A 58-year-old chronic smoker with history of recurrent pneumonia and bronchiectasis presented for septoplasty. Thoracic imaging revealed a dilated trachea and main bronchi, tracheal and bronchial diverticuli, and chronic bronchiectasis with mediastinal lymphadenopathy. An 8.5 cuffed endotracheal tube (ETT) proved too big for his glottic aperture. An 8.0 cuffed ETT with wet gauze packing yielding an adequate seal. Postoperative continuous positive airway pressure to prevent airway collapse followed awake extubation. Anesthetic concerns include grossly enlarged and weakened airways, inefficient cough mechanisms, presence of tracheal diverticuli, and post operative tracheal collapse. Anesthetic planning includes management of endotracheal cuff size. Small size yields air leak and ineffective ventilation. Large size may lead to mucosal damage. Tube dislodgement, copious secretions, chance of expiratory collapse due to the abnormally dilated and thin airways, and post operative monitoring all must be considered.

## 1. Introduction

Mounier-Kuhn syndrome or congenital tracheobronchomegaly is characterized by atrophy or absence of elastic fibers and smooth muscle cells leading to a markedly dilated trachea and bronchi associated with chronic pulmonary suppuration [1]. It clinically presents as chronic bronchitis or bronchiectasis, hence under diagnosed, and less than 100 cases are reported in the literature [2]. Our anesthetic management follows.

## 2. Case Description

A 58-year-old male (height: 5′ 2′′, weight: 131 lbs) presented for septoplasty. Significant past medical history included recurrent pneumonia, bronchiectasis, COPD necessitating home oxygen therapy, hypertension, benign prostatic hypertrophy, and adrenal insufficiency and 15-pack-year smoking history.

**Figure 1:**
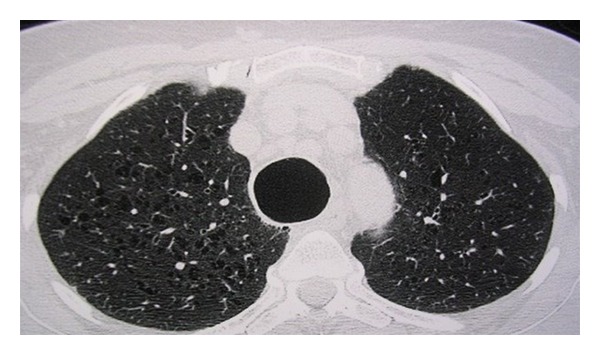


**Figure 2:**
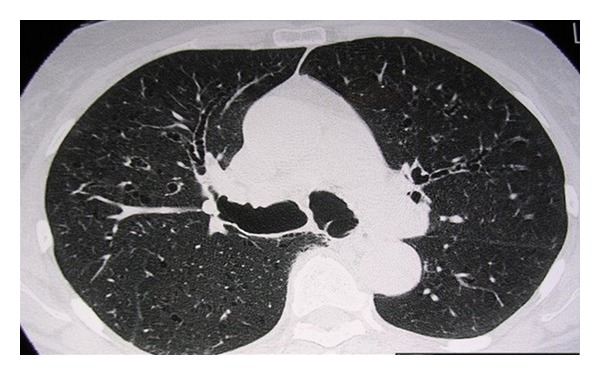


Chest X-ray revealed maximum transverse diameter of the trachea at 4.9 cm. Thorax computerized topography (CT) showed a dilated trachea and main bronchi (Figure 1), anterior tracheal diverticulum, left mainstem bronchial diverticulum, chronic bronchiectasis, and mediastinal lymphadenopathy presumably due to repeated respiratory infections (Figure 2).

Procedure planning included measuring the subglottic diameter of the trachea on the CT scan (1.54 × 1.48 cm) and measuring the diameter of different ET tubes with their cuffs inflated.

Premedication with glycopyrrolate 0.2 mg and hydrocortisone 100 mg IV preceded induction with propofol 150 mg. Tracheal intubation was facilitated with rocuronium 40 mg following confirmation of adequate ventilation. Initial intubation was done using a 8.0 ID cuffed endotracheal tube (ETT). Significant loss of volume was discovered, and decision to upsize to a 8.5 ID cuffed ETT was made. Following an unsuccessful attempt, secondary to the glottic opening being too small to accommodate the larger tube, it was decided to reinsert the 8.0 ID ETT and pack around the ETT until the air leak became acceptable. Packing was done with wet gauze using Magill's forceps just above the glottis around the ETT. Measured EtCO_2_ between 35 and 45 mmHg and *P*_peak_ 25 cm H_2_O guided ventilation. Procedural completion succeeded reversal with neostigmine and glycopyrrolate. At the time of extubation, ETT was suctioned yielding 200 mL purulent secretion. Standard extubation criteria were employed, extubated awake and transported to the PACU, kept on CPAP, and observed overnight prior to discharge.

## 3. Discussion

Idiopathic giant trachea bears the name of the clinician, Mounier-Kuhn, who originally described the radiographic and endoscopic appearance in a patient with recurrent lower respiratory tract infections in 1932 [3]. The tracheobronchomegaly is characterized by severe atrophy of longitudinal elastic fibers with thinning of the muscularis mucosa resulting in dilation of the membranous and cartilaginous portions of the trachea and bronchi. The trachea and bronchi are dilated with a transition to normal diameters in peripheral airways. Increased wall compliance allows the development of broad diverticulum like protrusions of redundant musculomembranous tissue between the cartilaginous rings [4]. The diagnosis can be made when the right mainstem, left mainstem, and trachea exceed 2.0, 1.8, and 3.0 cm, respectively, on chest CT [5].

Anesthetic plan must consider the balance between too little and too much air in the cuff; too little air results in air leak and hypercapnea too much air risks mucosal damage [6]. Other issues raised by this disorder include dislodgement of a mobile endotracheal tube [7], copious secretions, and chances of expiratory collapse of abnormally dilated and thin airways [8].

Messahel recommends use of an ETT with no cuff and charging the throat with wet gauze to reduce the leakage of anesthetic gas and danger of aspiration [6]. But Bourne et al. used ETT with a larger diameter with cuffs for the patient with abnormally large diameter of the trachea, because although cuff inflation is maximum, there is a danger of pulmonary aspiration due to severe air leakage around the cuff [9].

Accordingly we planned to use the largest diameter tube which would pass through the glottic opening and to inflate the cuff so as to prevent air leak and if necessary use wet gauze to reduce further leakage. Awake extubation after clearing out secretions and postoperative nasal CPAP is recommended [10]. This method proved successful for the management of a patient with this rare syndrome.

## References

[1] Lazzarini-de-Oliveira LC, Costa de Barros Franco CA, Gomes de Salles CL, de Oliveira AC (2001). A 38-year-old man with tracheomegaly, tracheal diverticulosis, and bronchiectasis. *Chest*.

[2] Kim MY (2010). Anesthetic experience of a patient with tracheomegaly—a case report. *Korean Journal of Anesthesiology*.

[3] Mounier-Kuhn P (1932). Dilatation de la trachee: constations radui-graphiques et bronchoscopiques. *Lyons Medical*.

[4] Himalstein MR (1973). Tracheobronchomegaly. *Annals of Otology, Rhinology and Laryngology*.

[5] Shin MS, Jackson RM, Ho KJ (1988). Tracheobronchomegaly (Mounier-Kuhn syndrome): CT diagnosis. *American Journal of Roentgenology*.

[6] Messahel FM (1989). Tracheal dilatation followed by stenosis in Mounier-Kuhn syndrome. A case report. *Anaesthesia*.

[7] Schwartz M, Rossoff L (1994). Tracheobronchomegaly. *Chest*.

[8] Woodring JH, Howard RS, Rehm SR (1991). Congenital tracheobronchomegaly (Mounier-Kuhn syndrome): a report of 10 cases and review of the literature. *Journal of Thoracic Imaging*.

[9] Bourne TM, Raphael JH, Tordoff SG (1995). Anesthesia for a patient with tracheobronchomegaly. *Anesthesia*.

[10] Collard P, Freitag L, Reynaert MS, Rodenstein DO, Francis C (1996). Respiratory failure due to tracheobronchomalacia. *Thorax*.

